# Roles of Protein Histidine Phosphatase 1 (PHPT1) in Brown Adipocyte Differentiation

**DOI:** 10.4014/jmb.1909.09003

**Published:** 2019-11-18

**Authors:** Joo Ae Kang Hyun Sup Kang, Kwang-Hee Bae, Sang Chul Lee, Kyoung-Jin Oh, Won Kon Kim

**Affiliations:** 1 Metabolic Regulation Research Center, KRIBB, Daejeon 344, Republic of Korea; 2 Department of Functional Genomics, University of Science and Technology (UST), UST-KRIBB School, Daejeon 34141, Republic of Korea

**Keywords:** Brown adipogenesis, histidine phosphorylation, obesity, PHPT1

## Abstract

Despite the importance of brown adipocytes as a therapeutic target for the prevention and treatment of obesity, the molecular mechanism underlying brown adipocyte differentiation is not fully understood. In particular, the role of post-translational modifications in brown adipocyte differentiation has not been extensively studied. Histidine phosphorylation is increasingly recognized an important process for protein post-translational modifications. In this study, we show that histidine phosphorylation patterns change during brown adipocyte differentiation. In addition, the expression level of protein histidine phosphatase 1 (PHPT1), a major mammalian phosphohistidine phosphatase, is reduced rapidly at the early phase of differentiation and recovers at the later phase. During white adipocyte differentiation of 3T3- L1 preadipocytes, however, the expression level of PHPT1 do not significantly change. Knockdown of PHPT1 promotes brown adipocyte differentiation, whereas ectopic expression of PHPT1 suppresses brown adipocyte differentiation. These results collectively suggest that histidine phosphorylation is closely linked to brown adipocyte differentiation and could be a therapeutic target for obesity and related metabolic diseases.

Protein post-translational modifications (PTMs) are an important mechanism for regulating a number of key intracellular processes, such as cell cycle, survival, proliferation or differentiation, by modulating protein function and signaling networks [1-3]. Virtually all proteins in eukaryotes undergo PTMs. There are more than 200 types of PTMs identified, but few have been subjected to in-depth studies. Due to technological improvement, especially MS-based proteomic analyses [4-6], not only the mapping of protein post-translational modifications are possible, but also PTM-related diseases have been continuously reported implying the importance of protein modifications in cellular metabolism.

Histidine phosphorylation is regarded as an important type of PTM in both prokaryotic and eukaryotic cells. On account of technical difficulties, such as the absence of phosphohistidine (pHis)-specific antibodies and the highly labile characteristics of pHis at low pH level or under heating condition, studies on histidine modification have been impeded [7]. However, the recent development of technology has enabled and facilitated the research on pHis. For example, Hunter *et al*. reported a total of 786 potential phosphohistidine substrates by proteomics analysis approaches using sequence-independent monoclonal pHis antibody [8]. In light of the fact that the number of potential substrates were much larger than expected, we hypothesized that histidine phosphorylation would be more critically associated with biological processes than previously thought.

There are only two histidine kinases are reported in mammalian cells so far, including NME1 (also known as nm23-H1 or nucleoside diphosphate kinase A, NDPKA) and NME2 (also known as nm23-H2 or nucleoside diphosphate kinase B, NDPKB) [9, 10]. These mammalian histidine kinases (NME1, NME2) are known to play important roles in cancer and tumor metastasis [11]. In addition, three histidine phosphatases, PHPT1 (phosphohistidine phosphatase 1), LHPP (phospholysine phosphohistidine inorganic pyrophosphate phosphatase), and PGAM5 (PGAM family member 5), have been further identified. It is reported that PHPT1 is involved in potassium ion transport and lipid metabolism. Additionally, PHPT1 regulates ATP-citrate lyase (ACLY) activity [12] or cell viability in neuroblastomas. It is also engaged in G-protein-mediated cell signaling in islet β cells [13]. However, there are only a few reports regarding the specific substrates of each histidine kinase and phosphatase. Thus, more detailed studies are necessary to understand the cellular mechanisms of histidine phosphorylation.

Obesity is caused by an imbalance between energy intake and expenditure, and increases the risk of metabolic disorders, such as type II diabetes and cardiovascular diseases. Many studies have been conducted to overcome obesity, and recent studies suggested the elevation of energy expenditure through the activation of brown adipocytes as a promising strategy [14]. Brown adipocytes consume fat as an energy source to produce heat by mitochondrial protein UCP-1 (uncoupling protein-1), which is exclusively expressed in brown adipocytes. As part of these attempts, studies on the role of post-translational modifications (PTMs) in brown adipocyte function or differentiation have been actively conducted, but were mainly focused on tyrosine phosphorylation, lysine acetylation, or methylation [2, 3, 15]. As such, the effects of histidine phosphorylation in brown adipocytes function or differentiation have not been identified yet. In this study, we investigated whether histidine phosphorylation affects brown adipocyte differentiation. We also analyzed the involvement of PHPT1, which regulates histidine phosphorylation during brown adipocyte differentiation.

To begin with, we checked the specificity of the anti-pHis antibody (Merck Millipore; MABS1341) in two mammalian cell lines using HEK293 (Human embryonic kidney 293 cell line) and C2C12 (Mouse skeletal myoblast cell line) [8]. As shown in Fig. 1A, distinct patterns were detected in HEK293 and C2C12 cells, and several bands disappeared after the heat treatment due to heat-sensitive characteristics of histidine phosphorylation.

To elucidate the functional role of histidine phospho-rylation on brown adipogenesis, we monitored histidine phosphorylation patterns of cell lysates without heat treatment during brown adipocyte differentiation. The immortalized brown preadipocyte cell line used in this study was kindly provided by Dr. Shingo Kajimura (UCSF, USA). Stromal vascular fraction cells isolated by collagen dispersion of interscapular brown adipose tissue from C57BL/6 mice at postnatal days 1-2 were differentiated to brown adipocytes, as described previously [16]. We previously reported the successful induction of these cells into mature brown adipocytes with the brown adipogenic markers of PGC-1α (peroxisome proliferator-activated receptor gamma coactivator 1 alpha), PRDM16 (PR domain containing 16), PPARγ (peroxisome proliferator-activated receptor gamma), and UCP-1 (uncoupling protein-1) [3, 17]. As shown in Fig. 1B, the pattern of histidine phospho-rylation changed significantly during the differentiation of the immortalized brown preadipocyte cell line. This pattern change could be explained by the balanced action between histidine kinases and phosphohistidine phosphatases. Therefore, we next examined PHPT1 expression changes during brown adipocyte differentiation. The expression level of PHPT1 was significantly decreased at an early stage of differentiation (day 2) and then recovered at a late phase of differentiation (Figs. 2A and 2B). These expression pattern changes suggest a functional role of PHTP1 during the early point of brown adipogenesis. We also checked the expression level of PHPT1 during white adipogenesis of 3T3-L1 cell line [18]. Unlike brown adipocyte differentiation, the PHPT1 expression levels did not change during white adipocyte differentiation (Figs. 2C and 2D), suggesting the specific role of PHPT1 in brown adipocyte differentiation.

To examine the functional roles of PHPT1 during brown adipogenesis, we depleted endogenous PHPT1 expression by a retroviral shRNA expression system (pSIREN-RetroQ-DsRed). As shown in Fig. 3A, three shRNA constructs against PHPT1 were examined [19]. Among the three shRNAs targeting PTPH1, shPHPT1-3 showed the most effective suppression of ectopically expressed FLAG-tagged PHPT1 in 293T cells (Fig. 3A). The shPHPT1-3 construct was co-transfected into GP2-293 with VSV-G plasmid, and the virus supernatant was collected and applied to immortalized brown preadipocytes. Cells expressing RFP were selected and enriched by FACS [20], and the stable suppression of endogenous PHPT1 was confirmed at both mRNA and protein levels (Figs. 3B and 3E). To investigate the function of PHPT1 in brown adipocyte differentiation, PHPT1 knockdown cells were induced to differentiate for six days. PHPT1 knockdown promoted differentiation leading to increased lipid droplet accumulation in comparison to that of control cells (Fig. 3C). Additionally, essential brown adipogenic markers, specifically PGC1α, PRDM16, PPARγ, and UCP1, were up-regulated upon the knockdown of PHPT1 (Figs. 3D and 3E). Moreover, histidine phosphorylation of ACLY, a known target of PHPT1, was increased in PHPT1-depleted mature brown adipocytes (Fig. 3F), suggesting that ACLY histidine phosphorylation might play an important role in brown adipogenesis.

To further clarify the roles of PHPT1 in brown adipo-genesis, we analyzed the effect of the ectopic expression of PHPT1 during brown adipocyte differentiation. Immortalized brown preadipocytes were infected with PHPT1 over-expressing retroviruses (PHPT1-IRES-GFP) and the infected cells were enriched by FACS sorting [20]. As shown in Fig. 4A, most of the cells were observed to be GFP-positive under fluorescence microscopy. Moreover, the ectopic expression of PHPT1 was confirmed by checking PHPT1 mRNA and protein levels (Fig. 4B). Contrary to the PHPT1 knockdown result, ectopic expression of PHPT1 showed decreased brown adipocyte differentiation, as measured by a lower degree of lipid droplet accumulation (Fig. 4C). Brown adipogenic genes also showed the reduced expression levels (Figs. 4D and 4E). Additionally, ACLY histidine phosphorylation was inhibited by PHPT1 overexpression in brown adipocytes (Fig. 4F), implying that PHPT1 might regulate brown adipocyte differentiation by modulating histidine phosphorylation of ACLY. However, further studies are needed to elucidate the detailed mechanism between histidine phosphorylation of ACLY and brown adipogenic differentiation. Previously, Seeger *et al*. reported cytotoxicity induced by the overexpression of PHPT1 in HUVEC cells (human umbilical-vein endothelial cells). Therefore, we examined the effect of the ectopic expression of PHPT1 on the viability of brown preadipocytes and mature brown adipocytes, [17]. However, the ectopic expression of PHPT1 in both brown preadipocytes and differentiated brown adipocytes had no significant effects on cell viability under our experimental conditions (Fig. 4G). These results indicate that the decreased differentiation efficiency is not due to decreased cell viability.

In this study, we observed protein histidine phosphorylation pattern changes during brown adipocyte differentiation, suggesting that pHis may be involved in brown adipogenesis. Second, PHPT1 is suppressed at an early phase and it recovers at a later stage of differentiation, implying the involvement of PHPT1 in brown adipocyte differentiation through the modulation of the protein histidine phosphorylation. Third, both the knockdown and ectopic expression of PHPT1 had significant effects on brown adipocyte differentiation. Although the current studies were limited to PHPT1 among the three phosphohistidine phosphatases and the direct target substrate(s) of PHPT1 remain to be identified, our results reveal a significant role for protein histidine phosphorylation and PHPT1 during brown adipocyte differentiation.

## Figures and Tables

**Fig. 1:**
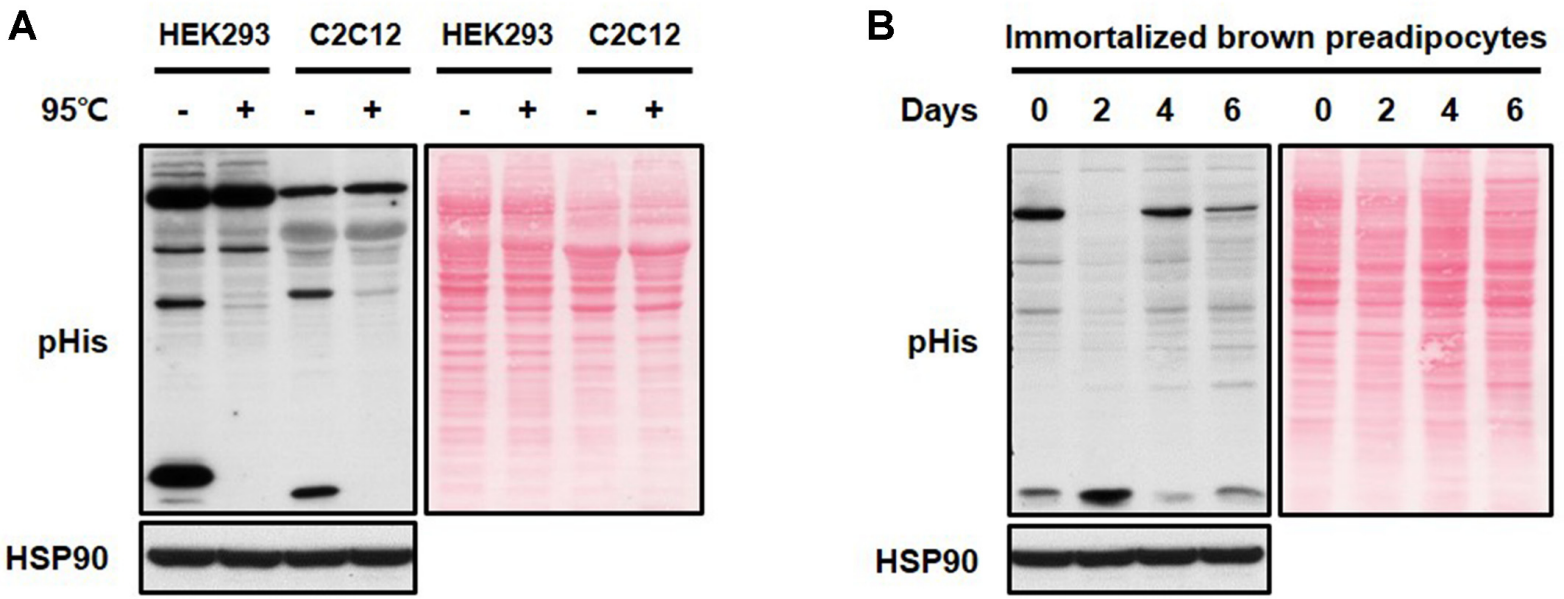
The pattern of protein histidine phosphorylation changes during brown adipocyte differentiation. (**A**) The expression patterns of protein histidine phosphorylation in HEK293 cells and C2C12 cells. Cell lysates with (+) or without (-) heat treatment at 95°C for 10 min. (**B**) The expression patterns of protein histidine phosphorylation during brown adipocyte differentiation without heat treatment. Whole-cell lysates were harvested at the indicated days of differentiation, and analyzed with pHis antibody. Western blot showing the pHis pattern (left), and Ponceau S staining of the membrane shown the protein patterns (right). HSP90 was used as a loading control.

**Fig. 2:**
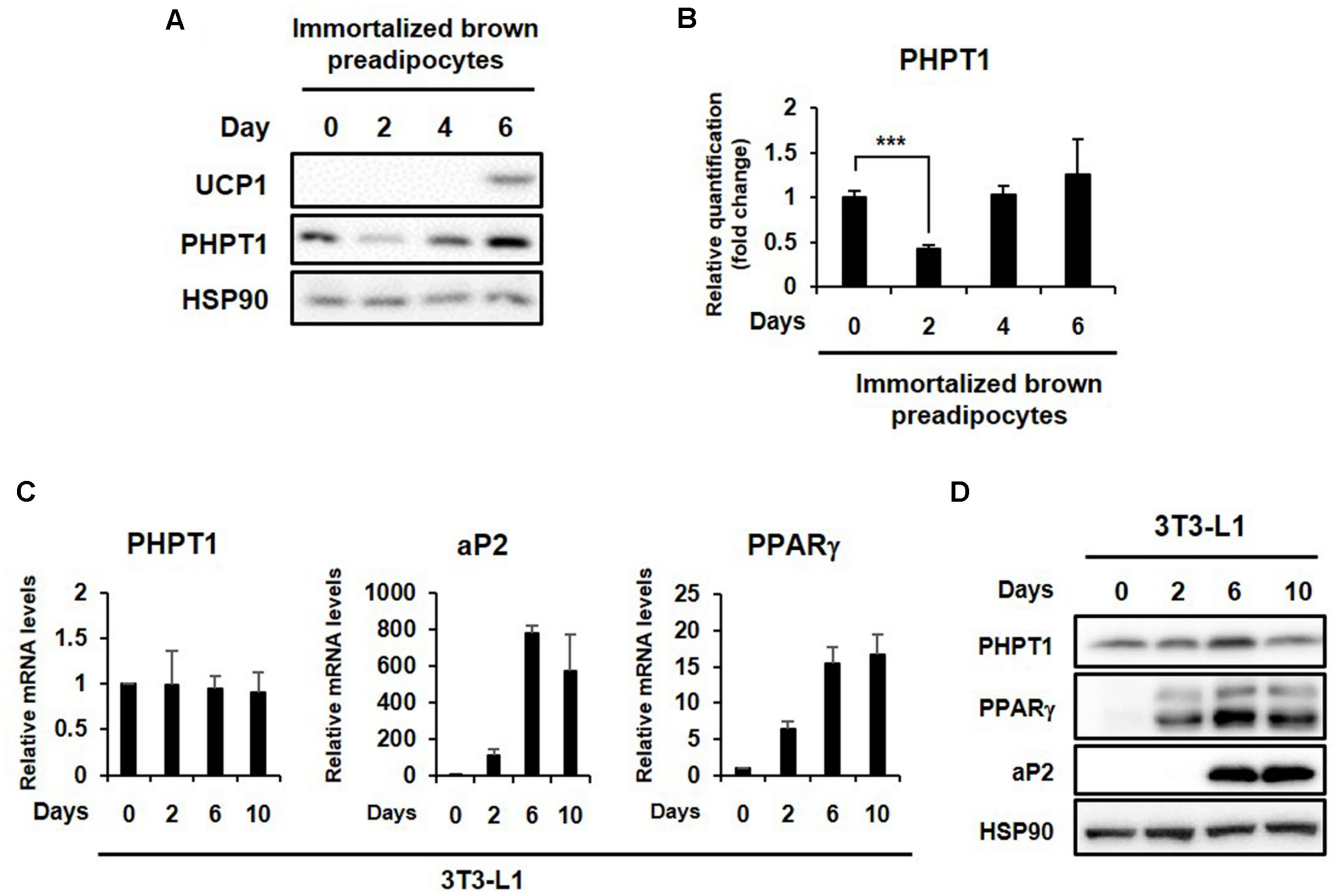
The expression levels of PHPT1 during brown or white adipocyte differentiation. (**A**) PHPT1 protein expression and (**B**) a quantitative analysis during brown adipocyte differentiation. The data represent the means ± SD (*n* = 3). Statistical analysis was performed using an independent Student’s *t*-test (***p < 0.0005). (**C**) PHPT1 mRNA expression (**D**) and protein expression during 3T3-L1 white adipogenesis were examined. Total RNA and protein were extracted on the indicated days of differentiation. The mRNA expression levels were normalized to TATA-box binding protein (TBP).

**Fig. 3:**
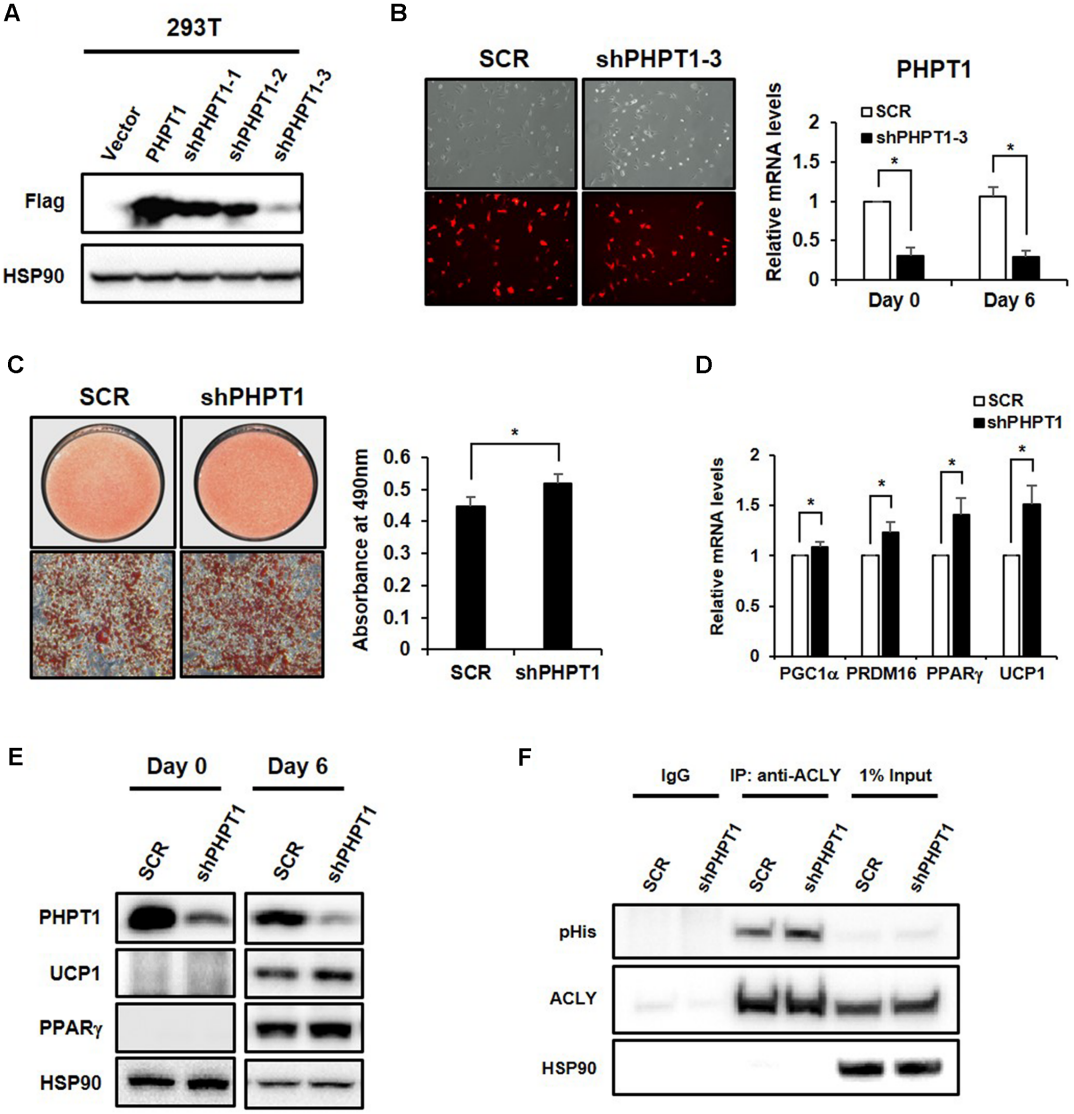
PHPT1 suppression promotes brown adipocyte differentiation. (**A**) The efficiency of knockdown construct targeting PHPT1 in 293T cells expressing FLAG-tagged PHPT1 examined by western blot analysis. The FLAG-tagged PHPT1 was ectopically expressed in 293T cells followed by transfection with three different PHPT1 shRNA constructs. (**B**) The most efficient shPHPT1-3 construct was infected into immortalized brown preadipocytes by retrovirus system (pSIREN-RetroQ-DsRed). Also, RFP expression and knockdown efficiency was checked. (**C**) Oil-Red-O staining of lipid droplets in PHPT1-depleted mature brown adipocytes (left) and quantitation of lipid content (right). Oil-red-O stain was eluted from cells with isopropanol, and measurements were taken at 490 nm. (**D**) The mRNA levels of brown adipogenic markers were determined by real-time PCR. (**E**) The corresponding protein levels of PHPT1, UCP1 and PPARγ were confirmed by a western blot analysis. (**F**) pHis on ACLY was analyzed in PHPT1 knockdown adipocytes. Total cell lysates containing equal amounts of protein were subjected to immunoprecipitation with anti-ACLY antibody. Western blot analysis was performed with anti-pHis, anti-ACLY, and anti-HSP90 antibodies. All quantitative data represent the means ± SD compared with control vector (SCR; scrambled shRNA, *n* = 3). Statistical analysis was performed using an independent Student’s *t*-test. *p < 0.05 vs. SCR.

**Fig. 4:**
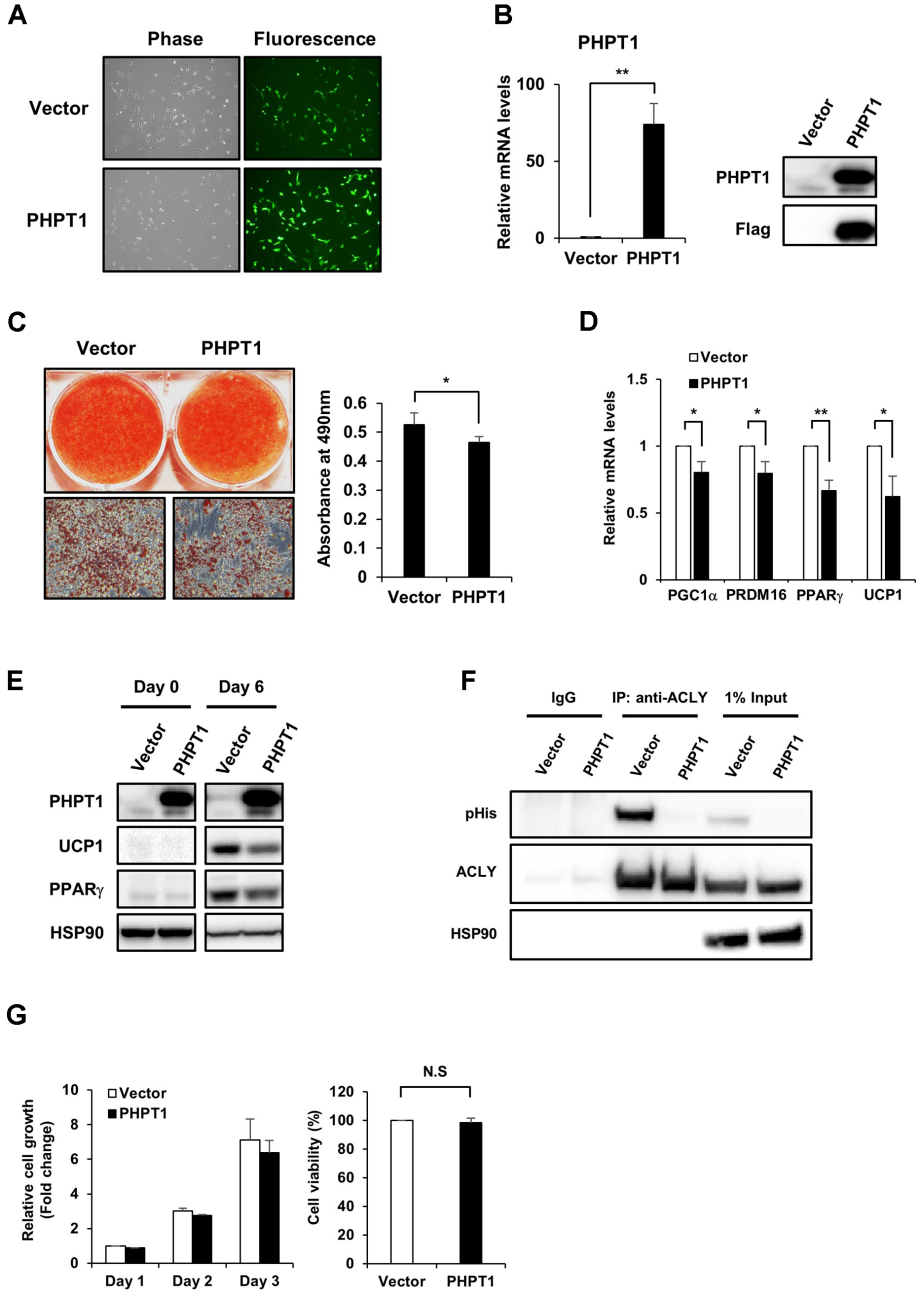
Ectopic expression of PHPT1 hampers brown adipocyte differentiation. (**A**) Immortalized brown preadipocytes were infected with retroviruses (pRetroX-IRES-ZsGreen1) expressing the control vector or FLAG-tagged PHPT1. The GFP expression was monitored by fluorescence microscopy. (**B**) The expression levels of PHPT1 in brown adipocyte cell lines were measured by real-time PCR and a western blot analysis. (**C**) Oil-Red-O staining of lipid droplets (left) and quantification of lipid accumulation (right) after differentiation was utilized to monitor the effect of the ectopic expression of PHPT1 on brown adipocyte differentiation. The method for quantification of lipid content was described in Fig. 3 legend. (**D**) The mRNA levels of brown adipogenic markers were measured by real-time PCR. (**E**) The corresponding protein levels of PHPT1, UCP1 and PPARγ were also analyzed by a western blot analysis at day 0 and day 6 after the induction of differentiation. (**F**) Analysis of histidine phosphorylation on ACLY in mature adipocytes overexpressing PHPT1. The method for immunoprecipitation is equal to that of Fig. 3 legend. The same membrane was re-probed and immunoblotted with the antibodies represented in the figure. Loading control was measured with anti-HSP90 antibody. (**G**) Cell viability was measured to investigate the effect of ectopic expression of PHPT1 in brown preadipocytes (left) and mature adipocytes (right), which showed no cytotoxic effects. The cell viability was measured using CellTiter-Glo Luminescent Cell viability assay kit (Promega). All quantitative data represent the means ± SD (*n* = 3). Statistical analysis was performed using an independent Student’s *t*-test. *p < 0.05 and **p < 0.005 vs. control vector.
